# Role of mast cells in the pathogenesis of severe lung damage in COVID-19 patients

**DOI:** 10.1186/s12931-022-02284-3

**Published:** 2022-12-21

**Authors:** Andrey V. Budnevsky, Sergey N. Avdeev, Djuro Kosanovic, Victoria V. Shishkina, Andrey A. Filin, Dmitry I. Esaulenko, Evgeniy S. Ovsyannikov, Tatiana V. Samoylenko, Alexander N. Redkin, Olga A. Suvorova, Inna M. Perveeva

**Affiliations:** 1 Department of Faculty Therapy, Burdenko Voronezh State Medical University, 10 Studencheskaya Str., Voronezh, Russia 394036; 2 Department of Pulmonology, I.M. Sechenov First Moscow State Medical University (Sechenov University), Healthcare Ministry of Russia, Trubetskaya Street 8, 119991 Moscow, Russia; 3 Scientific Research Institute of Experimental Biology and Medicine, Burdenko Voronezh State Medical University, Moskovsky Prospect, 185, Voronezh, Russia 394036; 4 Budgetary Health Care Institution of the Voronezh Region “Voronezh Regional Pathoanatomical Bureau”, Moskovsky Prospect, 151, Voronezh, Russia 394036; 5 Department of Pulmonology, Voronezh Regional Clinical Hospital, № 1, Moskovsky Prospect, 151, Voronezh, Russia 394036

**Keywords:** COVID-19, SARS-CoV-2, Mast cells, Inflammation, Cytokine storms

## Abstract

**Background:**

There is still insufficient knowledge with regard to the potential involvement of mast cells (MCs) and their mediators in the pathology of coronavirus disease-2019 (COVID-19). Therefore, our study aimed to investigate the role of MCs, their activation and protease profiles in the pathogenesis of early and late lung damage in COVID-19 patients.

**Methods:**

Formalin-fixed and paraffin embedded lung specimens from 30 patients who died from COVID-19 and 9 controls were used for histological detection of MCs and their proteases (tryptase, chymase) followed by morphometric quantification.

**Results:**

Our results demonstrated increased numbers of MCs at early stage and further augmentation of MCs number during the late stage of alveolar damage in COVID-19 patients, as compared to the control group. Importantly, the percentage of degranulated (activated) MCs was higher during both stages of alveolar lesions in comparison to the controls. While there was no prominent alteration in the profile of tryptase-positive MCs, our data revealed a significant elevation in the number of chymase-positive MCs in the lungs of COVID-19 patients, compared to the controls.

**Conclusions:**

MCs are characterized by dysregulated accumulation and increased activation in the lungs of patients suffering from COVID-19. However, future profound studies are needed for precise analysis of the role of these immune cells in the context of novel coronavirus disease.

## Clinical message

Our data shed the further light on the clinical issue of dysregulated lung inflammation, with particular focus on mast cells, in the context of COVID-19. This can provide an avenue for future development of targeted therapy against hyper-activated mast cells in order to treat COVID-19 patients.

## Introduction

Coronavirus disease 2019 (COVID-19) is a new infectious disease whose clinical manifestations range from asymptomatic to severe acute respiratory syndrome with widespread lung tissue damage that can cause mortality from this disease. Severe COVID-19 results in diffuse alveolar damage accompanied by the alveolar-hemorrhagic syndrome and widespread thrombosis of the microvasculature leading to hypoxia and respiratory failure [1, 2]. Lung pathology in COVID-19 is thought to be immune-mediated and exacerbated by the infiltration of monocytes, neutrophils, and subpopulations of T lymphocytes [3]. A cytokine storm causing much greater mortality than any direct viral cytotoxicity has been identified in about 15–20% of patients suffering from COVID-19 with severe lung damage [4]. COVID-19-associated cytokine storm is characterized by rapid proliferation and hyperactivation of the T-cell link, macrophages, and natural killer cells, as well as increased production of inflammatory cytokines and chemokines released by immune or non-immune cells [2, 5–8]. One of the sources of such cytokines and chemokines are mast cells (MCs), which are found everywhere in the body, especially in the organs of the respiratory system, and are crucial in the development of a number of pathological conditions [7, 9, 10].

MCs are a ubiquitous pool of cells of myeloid lineage strategically localized in the functionally significant areas of the body, including the skin, respiratory system, gastrointestinal mucosa, for optimal functional interaction with immunocompetent and stromal cells, as well as extracellular structures of a specific tissue microenvironment. MCs are represented by a highly heterogeneous cell population with subtype-dependent differences in morphology, histochemical properties, protease expression, and function. MCs have broad multifunctional properties associated with the release of many preformed mediators, phagocytosis, antigen processing, cytokine production, etc. [11, 12]. They can also recruit immune cells into the focus of inflammation, secreting chemokines and other mediators that can locally increase vascular permeability [12]. Histamine released by MCs in combination with interleukin (IL)-1 can cause increased lung inflammation resulting from severe acute respiratory syndrome coronavirus 2 (SARS-CoV-2) viral infection [13]. Certain mediators derived from MCs, proteases, such as β-tryptases, chymases, and carboxypeptidase A (CPA)-3 are considered specific for MCs and indicate the state of their activation [12, 14]. The functionality of serine proteases, especially tryptases and chymases, has led to an interest in studying the regulatory effects on the course of coronavirus infection. For example, it is known that chymase engages in the active hydrolysis of angiotensin I to angiotensin II, thereby being a potential participant in the development of heart failure and pulmonary hypertension [15].

Importantly, scientists in many countries are actively studying pathomorphology of the lung lesions in COVID-19, but only a few studies are devoted to the role of MCs in the pathogenesis of the disease. Therefore, our study objective was to assess the protease profile of MCs and the role of their activation in the pathogenesis of early and late lung damage in the context of the novel coronavirus infection.

## Methods

This morphological study was carried out based on the materials of 39 autopsies of the lungs in the “Voronezh Regional Pathological Bureau” and approved by the local ethics committee of the Burdenko Voronezh State Medical University (protocol №8, September 17, 2021). We studied autopsy lung specimens from 30 patients who died from COVID-19 and from 9 patients who died by accident or who died from other diseases in which the lungs were not pathologically affected (control group). This retrospective study included material only from those patients whose bodies were unclaimed by relatives after death (unclaimed corpses) and were subjected to municipal burial.

After sampling during autopsy and fixation in 10% neutral buffered formalin, paraffin embedding was carried out according to the standard sample preparation procedure [16]. From paraffin blocks, prepared sections with a thickness of 5 μm were used for staining with hematoxylin and eosin, Giemsa's dye, and picro Mallory. For immunohistochemical analysis, ultrathin sections, 2 µm thick, were prepared. Immunohistochemical staining was performed according to the standard protocol [16], detecting MC proteases—tryptase and chymase and evaluating the proliferation of type II alveolocytes by expression of PCNA. The identification of proteases was carried out using the primary mouse antibodies Anti-Mast Cell Tryptase antibody (clone AA1, #ab2378, dilution 1:4000) and Anti-Mast Cell Chymase antibody (#ab233103, dilution 1:1000), nuclear protein PCNA was detected using immunolabeling with primary rabbit monoclonal antibodies Anti-PCNA antibody (Epitomics #AC-0087RUO, clone EP91, dilution 1:1000, USA). Goat anti-rabbit and anti-mouse antibodies # AS-R1-HRP were used as secondary antibodies, which, in turn, visualized with ImmPACTTM DAB Peroxidase Substrat Kit (# SK-4105) according to the protocol specified in the instructions. The nuclei were contrasted with Mayer’s hematoxylin, after which the slices were placed in the mounting medium. The state of MC activation was assessed by the quantitative representation of tryptase and chymase-positive mast cells [9–11].

The stained micro-preparations were studied on a ZEISS Axio Imager.A2 microscope with a photo documentation system equipped with a Camera Axiocam 506 color digital camera. The images were processed using ZEN 2.3 software (Carl Zeiss, Germany). The total number of MCs was counted on the × 40 lens with the analysis of at least 50 fields of view. Planimetric analysis of micro-preparations included counting of MCs metachromatically stained with Giemsa dye, followed by functional distribution according to the degree of degranulation, and quantitative analysis of the protease profile (tryptase, chymase) per mm^2^.

The results were statistically processed using STATGRAPHICS Centurion XV software package. The normality of data distribution was assessed using normalized kurtosis and skewness coefficients. Quantitative data under normal distribution were presented as mean ± SEM, where SEM was standard error of the mean. If the sample did not meet the criteria for normal distribution, the data were presented as a median; the upper and lower quartiles were indicated in brackets. In the case of normal distribution, the comparison of variables was carried out using a one-way analysis of variance (ANOVA). If variables did not meet the criteria of normal distribution, the comparison was carried out using the Mann–Whitney U-test. p-value < 0.05 was considered significant.

## Results

Between September 21 and November 30, 2021, thirty patients with confirmed SARS-CoV-2 infection were included in the study. Twenty-one (70%) of patients were men, nine (30%) were women, and all patients were Caucasian. The mean age at death was 66.1 ± 15.7 years. Diabetes mellitus (70%), chronic kidney disease (50%) and coronary heart disease (37%) were the most common comorbidities. The majority of patients were obese (80%), with a mean body-mass index (BMI) 31.1 ± 4.9 kg/m^2^. All patients (100%) had acute respiratory distress syndrome (ARDS), were intubated and received invasive mechanical ventilation. The mean time of respiratory support was 10.4 ± 1.9 days. All patients (100%) were treated with systemic steroids. The mean duration of hospitalization was 12.5 ± 2.7 days, and the mean interval from symptom onset to death was 22.7 ± 3.8 days. Baseline demographic and clinical characteristics are shown in Table 1.

**Table 1:** COVID-19 patients’ baseline demographic and clinical characteristics

Demographic parameters	
Male, n (%)	21 (70)
Female, n (%)	9 (30)
Age, years	66.1 ± 15.7
Clinical parameters	
BMI, kg/m^2^	31.1 ± 4.9
ARDS, n (%)	30 (100)
Maximal CRP, mg/L	240.2 ± 69.4
Duration of ICU stay, days	12.5 ± 2.7
Duration of IMV, days	10.4 ± 1.9
Systemic steroid administration, n (%)	30 (100)
Comorbidities	
Diabetes mellitus, n (%)	21 (70)
Coronary heart disease, n (%)	11 (37)
Chronic heart failure, n (%)	9 (30)
Obesity, n (%)	24 (80)
Chronic kidney disease, n (%)	15 (50)

Data are presented as absolute values (%) or mean ± SEM. *ARDS* acute respiratory distress syndrome, *BMI* body mass index, *CRP* C reactive protein, *ICU* intensive care unit, *IMV* invasive mechanical ventilation

Patients in control group (n = 9) had similar age (62.2 ± 6.8 years) and similar spectrum of comorbidities (obesity 67%, diabetes 56%, coronary heart disease 33%).

**Fig. 1:**
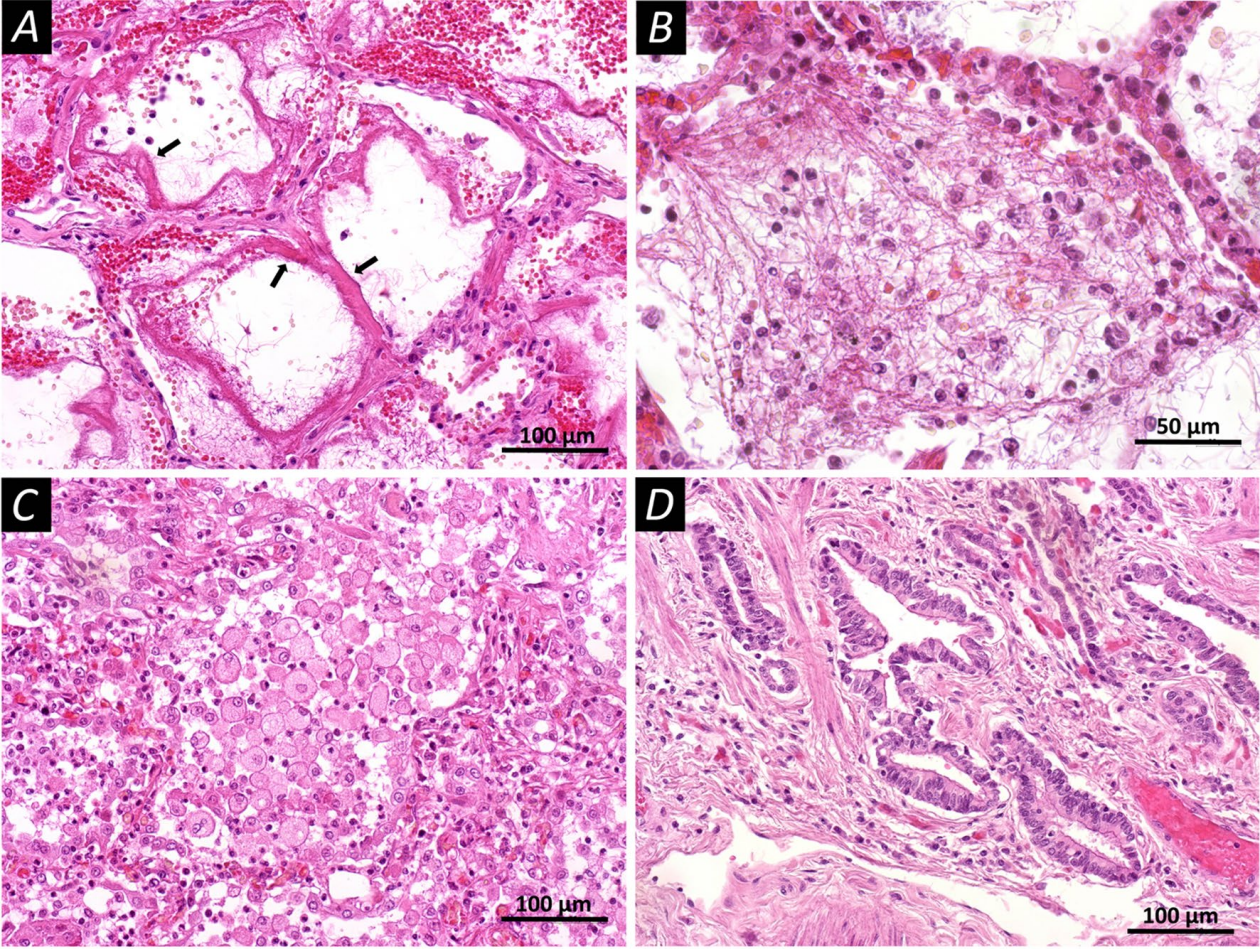
Microscopic changes in the lung tissues of patients who have died from COVID-19 (staining with hematoxylin and eosin). **A** Diffuse alveolar damage in the acute stage; the walls of the alveoli are filled with dense eosinophilic masses of fibrin, hyaline membranes (arrows); in the lumen of the alveoli, you can observe individual filaments of fibrin. **B** Diffuse alveolar damage in the acute stage, the lumen of the alveoli is filled with fibrin filaments, single erythrocytes, cells of the respiratory epithelium, and polymorphonuclear leukocytes. **C** Diffuse alveolar damage in the acute stage, round and oval cells almost completely filling the lumen of the alveoli. **D** Proliferation of type II alveolocytes leads to the formation of bizarre epithelial structures. Scale bars **A**, **C**, **D**—100 μm, **B**—50 μm

**Fig. 2:**
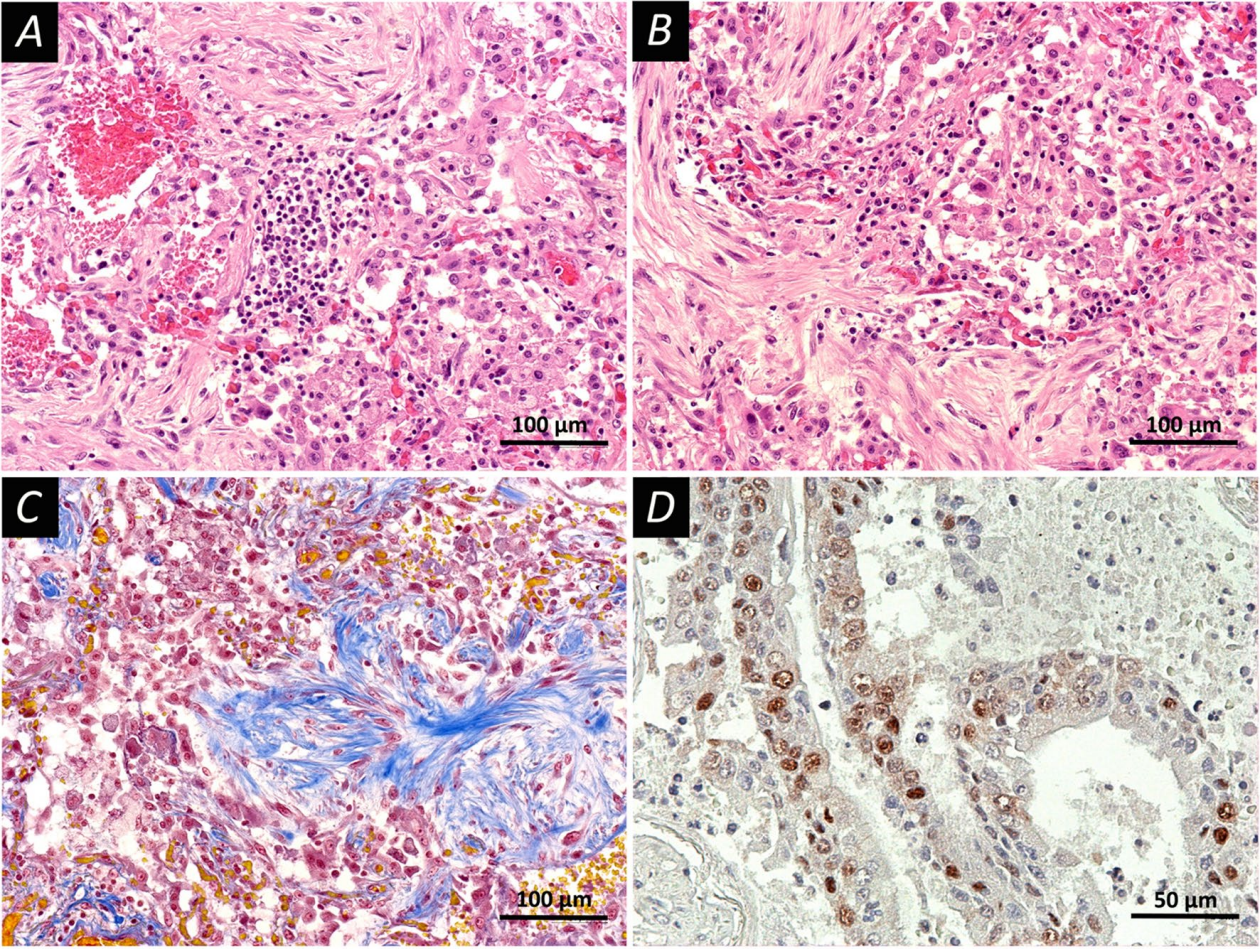
Late injury pattern in the lung tissue. Pronounced phenomena of the organization. Fixation: 10% neutral formalin. Technique: staining with hematoxylin and eosin (**A**, **B**) and picro Mallory (**C**) and PCNA was detected using immunolabeling with primary rabbit monoclonal antibodies Anti-PCNA antibody (Epitomics #AC-0087RUO, clone EP91, dilution 1:1000, USA), (**D**). **A** A late stage diffuse alveolar damage; the walls of the alveoli are thickened, hemorrhages and desquamation of the alveolar epithelium are observed, the focus of inflammatory infiltration is represented by lymphocytes. **B** A late stage diffuse alveolar damage; pronounced fibrosis of the alveolar septa, desquamation of the alveolar epithelium. **C** A late stage diffuse alveolar damage; special staining reveals the connective tissue fibers (blue color), focal hemorrhages, desquamation of the alveolar epithelium. **D** Expression of PCNA in type II alveolocytes (brown staining). Scale bars **A**–**C**—100 μm, **D**—50 μm

The early stage (Fig. 1) of diffuse alveolar damage (n = 15) was characterized by the following macroscopic changes: lung was light pasty to the touch, with areas of rubbery density on the rear surfaces, and reddish-violet on the surface. On the incisions, the lung tissue was grey-red; and a large amount of dark red foamy liquid was released from the surface of the incisions when pressed. Microscopic changes in this stage were represented by distelectasis, plethora of inter-alveolar septa, and accumulations of serous-purulent exudate in the lumens of the alveoli sporadically with an admixture of pinkish protein fluid with desquamated alveolocytes. Further, hyaline membranes were located parietally over a greater extent; the respiratory epithelium of the bronchial mucosa was desquamated and there were mixed blood clots in the lumens of some small branches of the pulmonary artery. The late stage (Fig. 2) of diffuse alveolar damage (n = 15) was macroscopically characterized by a dense-elastic consistency of the affected lungs, with a variegated surface due to alternation due to grey-pink, reddish-purple, and dark red areas. On incisions the lung tissue was airless with a complete loss of porous alveolar structures, greyish-reddish in color with dark red areas, a large amount of light red fluid is released from the surface of the incisions when pressed. Microscopically, in the stage of the late diffuse alveolar damage, there was pronounced vascular congestion of the inter-alveolar septa; there were erythrocytes with an admixture of macrophages in the lumens of the alveoli and proliferation of type II alveolocytes. Some alveoli contained filaments of fibrin, partly with the proliferation of fibroblasts, the beginning of the granulation tissue formation, and foci of squamous metaplasia of the alveolar epithelium. Multiple hyaline membranes lined the contours of the alveoli. Diffuse interstitial lymphoplasmacytic infiltration with an admixture of a small number of neutrophils was observed.

Analysis of histological preparations just on plain staining with hematoxylin and eosin, revealed an extensive representation of inflammatory cells in the affected tissues. These were diffuse and focal accumulations of lymphocytes, plasma cells, neutrophils, and eosinophils. Special methods made it possible to identify mast cells in the tissues of the affected lungs. They were also widely represented in all studied cases.

Control group samples presented numerous MCs. In most cases, the cells were localized in the perivascular spaces between the alveolar sacs and terminal bronchioles and in the alveolar septa, close to the alveolar capillaries (the result presented in Table 2). The percentage of MCs with signs of degranulation was 43.3% while 38.8% did not contain signs of degranulation. In control samples of lung tissue, mast cells formed autonomous, separately lying granules in 17.9%.

**Table 2:** The distribution of mast cells in the affected lung tissues of patients with early and late stages of alveolar damage (staining with Giemsa dye)

Stage of alveolar lesion	MC			dMC			nMC			Fragments		
	Early	Late	Control	Early	Late	Control	Early	Late	Control	Early	Late	Control
Average number of cells per mm^2^	27.7 ± 6.2	45.5 ± 10.1	13.4 ± 4.2	16.1 ± 4.4	25.9 ± 6.5	5.8 ± 2.4	0.8 ± 0.3	3.8 ± 1.9	5.2 ± 1.1	10.8 ± 2.6	15.8 ± 4.3	2.4 ± 0.8

Data are presented as mean ± SEM. *MC* mast cells, *dMC* degranulated mast cells, *nMC* non-degranulated mast cells, *Fragments* fragments of mast cells with granules

**Fig. 3:**
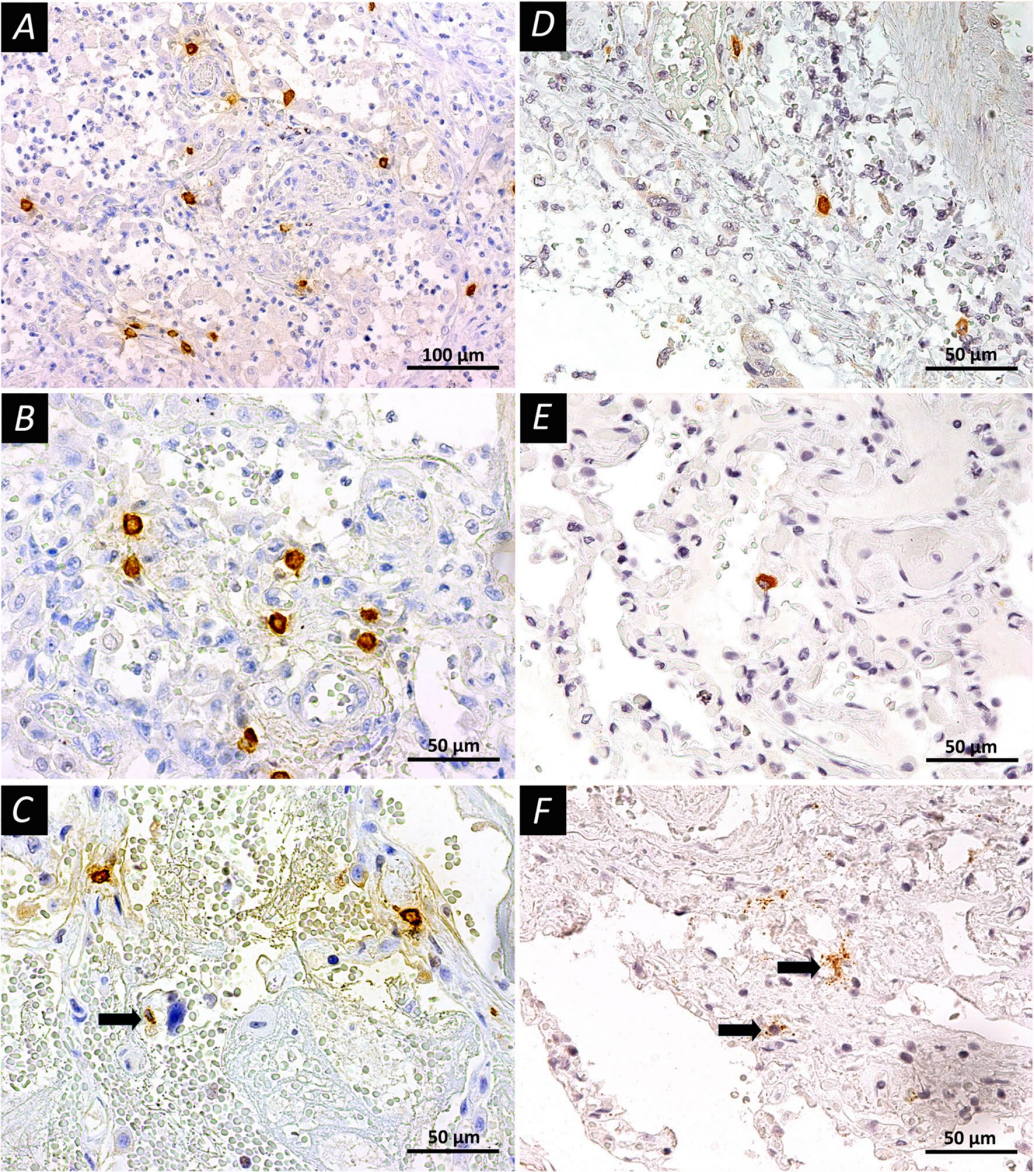
Mast cells in the lung tissues of COVID-19 patients. **A**–**C** Immunohistochemical staining with mouse monoclonal antibodies to tryptase (Abcam, # ab2378, dilution 1:4000) in accordance with standard protocol; **D**–**F** Immunohistochemical staining with mouse monoclonal antibodies to chymase (Abcam, #ab233103, dilution 1:1000). The peroxidase label on antibodies was visualized using DAB as a substrate (Vector Laboratories, Burlingame, CA, USA). The slices contrasted with Mayer’s hematoxylin. **A**, **B** Early diffuse alveolar injury, mast cell tryptase expression; mast cells are unevenly distributed in the alveolar septa. **C** Early diffuse alveolar damage, active degranulation of MCs, arrow indicates mast cell at the end of degranulation. **D**, **E** Uneven distribution of MCs with chymase expression. **F** Pronounced degranulation of mast cell chymase (arrows) with alveolar damage in the late stage. Scale bars **A**—100 μm, others—50 μm

Evaluation of the distribution of MCs and their proteases in COVID-19 patients with different stages of alveolar lesions revealed some interesting features (Tables 2 and 3, Fig. 3). There was an increase in the number of MCs at early stage of alveolar damage (p = 0.042) and further augmentation of MCs number during the late stage of alveolar damage (p = 0.034), as compared to the control group. Furthermore, the percentage of degranulated MCs was higher during the early and late stages of alveolar lesions in comparison to the control group. The data obtained indicated a significant representation of MCs in the affected parts of the lungs. A high percentage of MCs with signs of degranulation indicated their activation and participation in the inflammatory process. Finally, we have also analyzed the MCs protease (tryptase and chymase) profiles in our samples. We found that there was no prominent alteration in tryptase-positive MCs between the control and COVID-19 groups. However, there was a significant augmentation in the number of chymase-positive MCs in the lungs of COVID-19 patients, compared to the controls. It is noteworthy that in this study, we found an interesting fact of the formation of clusters of degranulating MCs expressing tryptase and chymase in areas of the lung with hemorrhagic phenomena (Fig. 3), which may be related to the role of histamine as a mediator that aggravates the course of the disease.

**Table 3:** Protease profile (tryptase, chymase) and secretory activity of mast cells in the affected lung tissues

Parameter	COVID-19 group (n = 30)	Control group (n = 9)	p-value
Tryptase-positive MCs			
Non-Degranulated MCs	3.59 (2.36; 7.35)	2.00 (1.78; 2.44)	0.005
Degranulated MCs	22.46 (17.47; 36.89)	19.78 (15.78; 26.22)	0.309
Single MCs	23.34 (18.12; 37.78)	18.22 (16.89; 22.00)	0.167
Collaborative Diligence	1.97 (1.33; 3.28)	1.33 (0.89; 1.78)	0.029
Total	35.78 (24.0; 50.22)	28.00 (24.00; 35.11)	0.505
Chymase-positive MCs			
Non-Degranulated MCs	0.22 (0.00; 0.67)	1.11 (0.89; 1.11)	0.004
Degranulated MCs	9.07 (3.56; 13.33)	4.44 (2.00; 5.33)	0.039
Single MCs	7.95 (3.78; 12.74)	4.44 (3.11; 5.56)	0.161
Collaborative diligence	0.89 (0.00; 1.78)	0.00 (0.00; 0.44)	0.009
Total	15.53 (6.67; 25.87)	5.56 (3.56; 6.44)	0.014

Data are presented as median (the upper and lower quartiles). *MCs* mast cells

## Discussion

Overall, our study has demonstrated altered distribution of MCs in the affected lung tissues in COVID-19 patients with different stages of alveolar damage. In the studied preparations, particularly the late stage of alveolar damage was accompanied by an increase in both the total number of MCs per mm^2^ and other parameters, in comparison to the control lungs. Quantitative characteristics of the content of MCs and indicators of their activity (degranulation) corresponded to histological changes in the lung tissue in the early and late stages of alveolar damage. Therefore, at the early stage, patterns of alteration and exudation predominated, apparently taking place with the direct participation of MCs. In the later stages, patterns of proliferative inflammation began to join and the regulatory role of MCs increased, the fact supported by quantitative changes. It was also noted the formation of clusters of degranulating MCs expressing tryptase and chymase in areas of the lung with hemorrhagic phenomena (Fig. 3).

Histological examination of the lungs of patients who died from SARS-CoV-2 infection revealed extensive infiltration of immune cells, including MCs, in the interstitium and alveoli [7, 13, 17, 18]. Such a significant representation of MCs in the affected lung tissue certainly indicated their involvement in the pathological process. MCs activation by increased tryptase and chymase secretion in both mild and severe COVID-19-associated lung tissue lesions only confirms their active role in the pathogenesis of inflammation.

There are many mechanisms of MCs activation in a pathological process, and in the case of lung damage in COVID-19 patients, this question remains open. MCs are activated by allergens, but they can also be caused by molecular pathogens through the activation of Toll-like receptors (TLR) [7, 19]. Viruses can activate MCs through TLR [14] with a subsequent increase in expression of inflammatory mediators. Moreover, MCs can detect damage-associated molecular structures (DAMPs) from various types of viruses, and in the same way detect SARS-CoV-2 infection and respond to it.

Innate immune cells can be activated by DAMPs and pathogen-associated molecular structures (PAMP) in COVID-19 patients [19–24]. Some literature sources suggested that MCs may express angiotensin-converting enzyme 2 (ACE2), which is known as the main receptor for SARS-CoV-2, and serine proteases, including the cell surface transmembrane protease serine 2, which are required to prime the coronavirus spike protein [7, 18, 19, 24, 25]. Conversely, the recent findings do not indicate the detectable levels of ACE-2 expression in the bone marrow MCs [26]. Therefore, this scientific question remains to be ultimately resolved in the future research. Moreover, MCs express a number of serine proteases that may be linked to the pathology of infection with SARS-CoV-2 [3, 9, 10]. Human MCs can be synergistically stimulated by peptide substance P and IL-33 to release a significant amount of vascular endothelial growth factors, IL-1β, or tumor necrosis factor without secretion of histamine or tryptase [23, 27]. SARS-CoV-2 induces the production of IL-1 by relevant cells, including MCs, causing induction of gene expression and activation of other pro-inflammatory cytokines. Since IL-1 is toxic, its production from MCs and macrophages activated by SARS-CoV-2 can also trigger gastrointestinal and brain disorders [13, 21]. Histamine is stored endogenously within the secretory granules of MCs and is involved in the increased expression of chemokine IL-8 and cytokine IL-6, thus favoring the hyper-inflammation in the lung. Therefore, in the context of COVID-19 cytokine storm and severe disease, histamine produced by MCs may induce the appearance of the microvascular leakage, proteases, and IL-6 that can degrade matrix. This may subsequently lead to the intra-alveolar formation of the hyaline membrane and perpetuation of inflammation, with release of angiogenic factors and pro-coagulative factors that may trigger immune thrombosis [28]. In the lung samples, we observed chymase-positive MCs with signs of active degranulation in the areas of damaged tissues, characterized by edema, hemorrhages and necrosis. It is known that chymase activates transforming growth factor-β (TGF-β) and matrix metalloproteinase (MMP) 9, converting their precursors into active forms involved in inflammation and fibrosis of tissues, which leads to structural damage and remodeling of organs, as well as to increased coagulation, which may be important in the pathogenesis of COVID-19 [7, 11, 21–23, 25, 29, 30].

The indication that hyper-activated MCs and the developing cytokine storm contribute to the severe course of COVID-19 is described [12, 25, 31, 32]; this fact suggests new possibilities (approaches) in the pharmacotherapy of both the acute process and post-COVID-19 period. However, some studies provided a contrasting data with regard to the topic of the involvement of MCs in the pathology of COVID-19. Schaller et al. found differences in the MCs numbers between early and late stages of the lung damage, suggesting that MCs may play a role in the later stages [33]. In addition, Giannetti and colleagues concluded that MCs activation is not important phenomenon in the context of COVID-19 [26].

## Conclusions

The study of the role of MCs in COVID-19 associated lung damage can shed light on the specifics of the pathogenesis of this disease, the severity of its manifestation, and the mechanisms of development of fatal cases. In addition, the involvement of MCs in the pathological process may give an opportunity to develop targeted therapy for COVID-19 patients by pharmacological interference with MCs activation (degranulation) and inhibition of their mediators. Finally, the determination of proteins specific for MCs in biological fluids may allow us to predict the clinical course in a particular patient. However, all these future possibilities require further and profound study of this important scientific and clinical issue.

## Data Availability

Data are available from the corresponding author upon the reasonable request.
